# Structural and Physiological Exploration of *Salmonella* Typhi YfdX Uncovers Its Dual Function in Bacterial Antibiotic Stress and Virulence

**DOI:** 10.3389/fmicb.2018.03329

**Published:** 2019-01-14

**Authors:** Hye Seon Lee, Soohyun Lee, Jun-Seob Kim, Hae-Ran Lee, Ho-Chul Shin, Moo-Seung Lee, Kyeong Sik Jin, Cheol-Hee Kim, Bonsu Ku, Choong-Min Ryu, Seung Jun Kim

**Affiliations:** ^1^Disease Target Structure Research Center, Korea Research Institute of Bioscience and Biotechnology, Daejeon, South Korea; ^2^Department of Biology, Chungnam National University, Daejeon, South Korea; ^3^Infectious Disease Research Center, Korea Research Institute of Bioscience and Biotechnology, Daejeon, South Korea; ^4^Pohang Accelerator Laboratory, Pohang University of Science and Technology, Pohang, South Korea; ^5^Department of Biotechnology, University of Science and Technology KRIBB School, Daejeon, South Korea; ^6^Department of Bioscience, University of Science and Technology KRIBB School, Daejeon, South Korea

**Keywords:** STY3178, YfdX, *Salmonella* Typhi, antibiotics susceptibility, virulence

## Abstract

YfdX is a prokaryotic protein encoded by several pathogenic bacteria including *Salmonella enterica* serovar Typhi, which causes one of the most fatal infectious diseases, typhoid fever. YfdX is a product of the *yfdXWUVE* operon and is known to be under the control of EvgA, a regulator protein controlling the expression of several proteins involved in response to environmental stress, in *Escherichia coli*. Nevertheless, unlike other proteins encoded by the same operon, the structural and physiological aspects of YfdX have been poorly characterized. Here, we identified a previously unknown pH-dependent stoichiometric conversion of *S*. Typhi YfdX between dimeric and tetrameric states; this conversion was further analyzed via determining its structure by X-ray crystallography at high resolution and by small-angle X-ray scattering in a solution state and via structure-based mutant studies. Biologically, YfdX was proven to be critically involved in *Salmonella* susceptibility to two β-lactam antibiotics, penicillin G and carbenicillin, as bacterial growth significantly impaired by its deficiency upon treatment with each of the two antibiotics was recovered by chromosomal complementation. Furthermore, by using *Galleria mellonella* larvae as an *in vivo* model of *Salmonella* infection, we demonstrated that *Salmonella* virulence was remarkably enhanced by YfdX deficiency, which was complemented by a transient expression of the wild-type or dimeric mutant but not by that of the monomeric mutant. The present study work provides direct evidence regarding the participation of YfdX in *Salmonella* antibiotic susceptibility and in the modulation of bacterial virulence, providing a new insight into this pathogen’s strategies for survival and growth.

## Introduction

*Salmonella enterica* serovar Typhi is a gram-negative bacterium that infects humans only (Hurley et al., 2014), causing systemic typhoid fever. Typhoid is one of the most widespread and hazardous infectious diseases in developing countries, with more than 16 million cases and 200,000 estimated deaths per year (Buckle et al., 2012; Dougan and Baker, 2014; Azmatullah et al., 2015). *S.* Typhi initially penetrates the small intestinal epithelial cells and then spreads through the bloodstream to other organs such as the spleen, liver, and bone marrow, where this bacterium multiplies and reenters the bloodstream causing symptoms including a high fever (Everest et al., 2001). A variety of antibiotics, such as ampicillin, chloramphenicol, trimethoprim-sulfamethoxazole, and ciprofloxacin, have already been used against *S*. Typhi (Kalra et al., 2003). Nevertheless, exposure to antibiotics over long periods has allowed *S.* Typhi to acquire resistance to various antibiotics through genetic changes: a phenomenon called “multiple drug resistance (MDR)” (Rowe et al., 1997; Kalra et al., 2003; Frye and Jackson, 2013). MDR strains exhibit strong resistance to antibiotics, and lead to treatment failure (Rowe et al., 1997; Parkhill et al., 2001; Kariuki et al., 2015; Karkey et al., 2018).

Bacterial resistance to antibiotics is acquired via a combination of a variety of strategies, which include alteration of a target protein, enzymatic deactivation, and restriction of antibiotic accessibility (Nikaido, 2009). How bacteria orchestrate these complicated events accompanying a change in the expression of several proteins has been under intensive investigation. It has been reported that EvgA, one of the response regulator proteins of *Escherichia coli*, constitutes a two-component system together with the sensor kinase EvgS and controls the expression of a wide range of genes involved in the response to stressors such as antibiotics and pH changes (Nishino and Yamaguchi, 2001, 2002; Masuda and Church, 2002; Nishino et al., 2003). The *yfdXWUVE* operon is one of the targets upregulated by EvgA (Masuda and Church, 2002; Nishino et al., 2003) and encodes formyl-CoA transferase YfdW (Gruez et al., 2003), oxalyl-CoA decarboxylase YfdU (Werther et al., 2010), acetyl-CoA:oxalate CoA-transferase YfdE (Mullins et al., 2013), putative transporter protein YfdV, and an uncharacterized protein YfdX, whose expression has been reported to be enhanced a 1000-fold by EvgA overproduction (Nishino and Yamaguchi, 2002; Nishino et al., 2003). Homologs of YfdX have been identified in various virulent bacterial species including *S.* Typhi (Parkhill et al., 2001), *S.* Typhimurium (McClelland et al., 2001), *Hafnia alvei* (Lazaro-Diez et al., 2016), *Shigella dysenteriae* (Kaur et al., 2014), and *Klebsiella pneumoniae* (Jiang et al., 2010). Furthermore, the crystal structure of *K. pneumoniae* YfdX in the tetramer-estimated form was determined and deposited at the PDB without accompanying publications (PDB code 3DZA). Recently, it has been reported that a bacterial protein STY3178, an ortholog of YfdX from the *S.* Typhi representative MDR strain CT18, interacts with three antibiotics ciprofloxacin, ampicillin, and rifampin (Saha et al., 2016b), has a chaperone-like activity (Saha et al., 2016a), and is modeled to interact with the outer-membrane protein STY3179 (Mondal et al., 2017), providing clues for understanding this protein. However, a number of unresolved issues about YfdX remain, with the absence of structural and physiological analyses, including its functional role during bacterial infection as well as its precise stoichiometry. *K. pneumoniae* YfdX appears to form a homotetramer in the crystal structure, whereas *S.* Typhi YfdX is proposed to form a trimer in solution according to dynamic light scattering, size exclusion chromatography, and nuclear magnetic resonance experiments (Saha et al., 2016b).

In this study, we attempted to answer such unresolved issues about the YfdX protein through structural, biochemical, and physiological analyses. An unexpected stoichiometric conversion of *S.* Typhi YfdX between the dimer and tetramer was identified, which was further analyzed via mutational assays based on crystal structure determination. Moreover, we found that *Salmonella* YfdX plays a significant role in bacterial susceptibility to penicillin G and carbenicillin and is involved in the negative regulation of bacterial virulence in an insect larvae model. These data collectively expand our understanding of this poorly studied protein.

## Materials and Methods

### Crystallization and Structure Determination of *st_*YfdX

The DNA fragment coding for residues 10–186 of *st_*YfdX was cloned into the pET21a plasmid (Novagen). The protein was produced in the *E. coli* BL21(DE3) RIL strain (Novagen) at 18°C and purified on a Ni-NTA column (QIAGEN) first. The protein was further purified on a HiLoad 26/600 Superdex 75 prep grade gel filtration column (GE Healthcare), equilibrated with a buffer consisting of 20 mM Tris-HCl (pH 7.5), 200 mM NaCl, and 1 mM DTT. All crystals were obtained via the sitting-drop vapor diffusion method at 18°C by mixing and equilibrating 0.4 μL samples of the protein solution (20 mg/mL) with a precipitant solution as described in Table 1. Before data collection, crystals were immersed briefly in the reservoir solution containing a cryoprotectant reagent as shown in Table 1. Diffraction data were collected on the beamline 5C and 7A at the Pohang Accelerator Laboratory, South Korea, and processed using the *HKL* 2000 software (Otwinowski and Minor, 1997). The structure was determined by the molecular replacement method with the Phaser software (McCoy et al., 2007) using the structure of *kp_*YfdX (PDB code 3DZA) as a search model. Programs Coot (Emsley and Cowtan, 2004) and PHENIX (Adams et al., 2010) were used for the model building and refinement, respectively. Crystallographic data statistics are summarized in Table 2^1^.

**Table 1:** Crystallization and cryoprotectant reagent conditions.

	Space group	Crystallization condition	Cryoprotectant reagent
Crystal I	*F*222	0.1 M sodium acetate (pH 4.6), 2.5 M sodium chloride, 12% PEG1500, 1.5% 2-methyl-2,4-pentanediol	15% glycerol
Crystal II	*F*222	0.1 M sodium acetate (pH 4.6), 2.5 M sodium chloride, 12% PEG1500, 1.5% 2-methyl-2,4-pentanediol	5% glycerol
Crystal III	*P*222	4% Tacsimate (pH 5.0), 12% PEG3350	20% glycerol

**Table 2:** Data collection and structure refinement statistics.

PDB code	Crystal I (6A02)	Crystal II (6A07)	Crystal III (6A09)
Space group	*F*222	*F*222	*P*222
Unit cell dimensions			
a, b, c (Å)	88.8, 92.5, 95.5	88.8, 92,2, 95.4	72.4, 127.9, 170.4
α, β, γ (^o^)	90, 90, 90	90, 90, 90	90, 90, 90
Wavelength (Å)	0.9793	0.9793	0.9793
Resolution (Å)	50.0-1.4 (1.42-1.40)^b^	50.0-1.5 (1.53-1.50)^b^	50.0-2.3 (2.34-2.30)^b^
*R*_sym_^a^	9.5 (27.1)	8.1 (28.0)	8.0 (27.7)
*I*/σ(*I*)	41.3 (4.7)	38.3 (5.6)	29.5 (4.9)
Completeness (%)	98.8 (96.5)	99.4 (99.5)	98.5 (91.2)
Redundancy	9.4	6.0	5.5
**Refinement**			
Resolution (Å)	50.0-1.4 (1.43-1.40)	50.0-1.5 (1.54-1.50)	50.0-2.3 (2.35-2.30)
Number of reflections	38,242	31,146	70,194
*R*_work_^c^/*R*_free_	18.9/21.3 (28.2/29.3)	18.2/21.5 (22.2/23.5)	18.0/23.3 (23.1/27.6)
Number of atoms			
Protein/water and ion	1326/198	1337/223	10668/587
RMSD			
Bond lengths (Å)/angles (^o^)	0.005/0.713	0.005/0.720	0.008/0.839
Ramachandran plot (%)			
Favored/allowed	98.2/1.8	98.8/1.2	98.2/1.8
Average B-values (Å^2^)			
Protein/water and ion	17.1/27.2	19.1/30.0	37.4/38.7
Molprobity score	1.09 (99^th^ percentile; *N*= 3363, 1.400 ± 0.25 Å)	1.05 (99^th^ percentile; *N* = 4775, 1.501 ± 0.25 Å)	1.35 (100^th^ percentile; *N* = 8821, 2.293 ± 0.25 Å)

*^a^R_sym_= Σ |I_obs_ – I_avg_|/I_obs_, where I_obs_ is the observed intensity of individual reflection and I_avg_ is the average over symmetry equivalents. ^b^The numbers in parentheses are statistics from the shell with the highest resolution. ^c^R_work_ = Σ ||F_o_|– |F_c_||/Σ |F_o_|, where |F_o_| and |F_c_| are the observed and calculated structure factor amplitudes, respectively. R_free_ was calculated with 10% of the data.*

### Preparation of Recombinant Proteins

Each of the DNA fragments encoding mutant *st_*YfdX proteins containing F42A^⋅^F45A^⋅^Y165A or Y100A^⋅^I137A substitutions and a DNA fragment encoding *kp*_YfdX was cloned into the pET21a plasmid. The recombinant proteins were produced and purified as was the wild-type *st_*YfdX.

### Size Exclusion Chromatography-Multiangle Light Scattering Experiments

Size exclusion chromatography-multiangle light scattering (SEC-MALS) was performed using Superdex 75 Increase 10/300 GL (GE Healthcare). The differential refractive index spectra were recorded on Optilab T-rEX (Wyatt Technology Corporation), which was combined with high-performance liquid chromatography (Shimadzu) and DAWN HELEOS-II (Wyatt Technology Corporation). The weight-average molar mass was calculated using the ASTRA 6 software (Wyatt Technology Corporation).

### Circular Dichroism Spectroscopy

Data were collected on a JASCO model J-815 spectropolarimeter with a 0.1-cm cuvette. The circular dichroism (CD) spectrum was recorded over the range of 200–260 nm in a nitrogen atmosphere with 0.1 mg/mL protein samples dissolved in 40 mM sodium phosphate buffer (pH 6.0 or 7.5). The spectrum comprised the accumulation of three scans at 0.2 nm intervals, which was corrected by subtracting signals from the buffer control. The raw CD signals were converted to mean residue ellipticity [*𝜃*_MRE_] (in deg cm^2^ dmol^-1^) using the equation [*𝜃*_MRE_] = *𝜃*_obs_/*Cnl*, where *𝜃*_obs_ is the observed ellipticity (in millidegrees), *C* is the protein concentration (in molarity), *n* is the number of amino acid residues, and *l* is the pathlength (in millimeters).

### Small-Angle X-Ray Scattering Experiments

Small-angle x-ray scattering (SAXS) measurements were carried out using the 4C SAXS II beamline at the Pohang Accelerator Laboratory, South Korea (Kim et al., 2017). Data were collected at 4°C with a sample-to-detector distance of 4,000 and 1,000 mm. Protein samples were prepared at pH 5.5 and 8.0, each of which was diluted to three different concentrations (1, 3, and 5 mg/mL). The SAXS data of each sample were collected in 10 successive frames of 5 s at 0.734 Å wavelength, which were measured in triplicates. Two-dimensional SAXS patterns were averaged and normalized for further analysis. Scattering intensities from the buffer solution were used as the experimental background. The scattering intensity data *I*(*q*) as a function of q (q = 4πsin𝜃/λ, where 𝜃 is half of the scattering angle and λ is the wavelength; 0.01 Å^-1^ < q < 0.7 Å^-1^) were obtained by radial averaging. CRYSOL (Svergun et al., 1995) and GNOM (Semenyuk and Svergun, 1991) were used to calculate the SAXS curves and distance distribution function *P*(*r*), respectively. Molecular envelops were reconstructed using the *ab initio* shape determination program DAMMIF (Franke and Svergun, 2009). Fifteen independent models were generated, compared, and averaged to obtain the refined models using the programs DAMAVER and DAMSTART. Surface rendering was achieved using the program PyMOL. Structural diagrams were superimposed onto the reconstructed dummy atoms using SUPCOMB (Kozin and Svergun, 2001). Normalized spatial discrepancies were 3.13 for the tetramer and 3.32 for the MolA–Mol B dimer.

### Knockout and Complementation of *yfdX*

*Salmonella* transformants carrying the pKD46 Red helper plasmid were cultured in the Luria-Bertani (LB) medium containing 100 μg/mL ampicillin and 10 mM L-arabinose at 30°C until turbidity at 600 nm reached 0.35–0.4. The cells were harvested and washed three times with ice-cold 10% glycerol and sterilized water to prepare electro-competent cells. A polymerase chain reaction (PCR) product containing the kanamycin resistance gene (amplified from pKD4) flanked upstream and downstream by sequences of the target gene (*yfdX*) was obtained using primers *yfdX*-p1 (5′-TGGCCGCAACAAACATGACTGATAACGTTACTCTGAATAATGACAAGATGTGTAGGCTGGAGCTGCTTC-3′) and *yfdX*-p2 (5′-GCGCGGCGTCGTGCTGCACGGAGTGCGTGGGTGATTCCTGAACTGACTGAATGGGAATTAGCCATGGTCC-3′). Next, 50 μL of electro-competent cells was mixed with 500 ng of the PCR product containing the 50 bp homologous sequence arms at each end of the kanamycin resistance cassette. Electroporation was performed in a 2-mm cuvette on Gene Pulser 3 (Bio-Rad) set at 2.5 kV, 25 μF, and 200 Ω. Transformants were screened on LB agar containing 50 μg/mL kanamycin. Knockouts were confirmed with primers *yfdX*-up97 (5′-GTGGGTTACCGGTTCTGAATAG-3′) and *yfdX*-dn43 (5′-CATAATGCTGCCTGCTGTAATG-3′). The kanamycin cassette was removed by pCP20 transformation (Datsenko and Wanner, 2000). For chromosomal complementation, the *yfdX*-kanamycin resistance cassette fusion PCR products were prepared using primers *yfdX*-ATG20 (5′-ATGGTTATCCTGTTTTCAGG-3′) and *yfdX*-dn_H_P2 (5′-TGTCAGACATAATGCTGCCTGCTGTAATGTATACAGCAGGCTGGGGGATAATGGGAATTAGCCATGGTCC-3′). Homologous recombination was performed as described above. For plasmid complementation, *yfdX* genes encoding wild-type or each mutant were cloned into the pNM12 vector harboring the *ara* promoter and ampicillin marker, which were then transformed into the *S*. Typhimurium UK-1 Δ*yfdX* strain.

### Phenotype Microarrays

BiOLOG phenotypic microarray analyses were conducted following the manufacturer’s instructions (BiOLOG, Inc.). In brief, bacteria were grown at 37°C for 16 h on LB agar media, and then scraped and resuspended in IF-0a. After that, 100 μL of a cell resuspension was inoculated into each well of phenotype microarray plates (#1–20). Respiration signals from 0 to 24 h shown in red were measured by BiOLOG OmniLog and then compared between the two strains. The microarray results were validated following the BiOLOG protocol with small modifications. To determine the minimum inhibitory concentration, serially diluted antibiotics were mixed with cell suspensions (5 × 10^5^ cfu/mL), and then respiration signals were measured. All the experiments were conducted in triplicate.

### Insect-Toxicity Assay

Injection assays with *Galleria mellonella* larvae were performed as described by Flury et al. (2016) with small variations. *G. mellonella* caterpillars purchased from S-WORM were grown at 30°C until they reached the fourth larval instar. Caterpillars were stored in the dark at 30°C for 3 days for stabilization. For injection, suspensions of overnight cultured bacterial cells were prepared in pH 7.4 PBS consisting of 137 mM NaCl, 2.7 mM KCl, 4.3 mM Na_2_HPO_4_, and 1.47 mM KH_2_PO_4_, and diluted to the desired concentration (10^5^ cfu/μL). Bacterial cells resuspended in 2 μL of PBS were injected into larvae with a 5 μL micro-syringe (MicroliterTM #701, Hamilton). The larvae were kept in Petri dishes at 30°C in the dark. For the evaluation of bacterial virulence, larvae were scored as live or dead for every 6 h up to 72 h. Each experiment consisted of three replicates per treatment with 10 larvae per replicate.

### Bacterial RNA Isolation and Real-Time PCR

*Salmonella* Typhimurium UK1 strains were grown in the LB medium under aerobic conditions at 37°C for 4, 8, and 12 h before RNA isolation. The cells were stabilized using the RNA protect Bacteria Reagent (Qiagen), and total RNA was isolated using the RNeasy Kit (Qiagen). cDNA was synthesized with Superscript III Reverse transcriptase (Invitrogen). The mRNA levels of the coding regions of the *yfdX* gene were measured by quantification of cDNA using SYBR Green PCR Master Mix (Bio-Rad) with primers Q-ST-yfdX-F (5′-CTAGCAGGCGTCAGTGTTATT-3′) and Q-ST-yfdX-R (5′-ATGCCATCCTGAGCTGATTT-3′) monitored on a CFX connect real-time PCR detection system (Bio-Rad). The mRNA levels of the *yfdX* gene were normalized to the levels of 16S ribosomal RNA amplified with primers ST-16S-QF (5′-GTTAGCCGGTGCTTCTTCTG-3′) and ST-16S-QR (5′-TAGGCCTTCGGGTTGTAAAGT-3′).

## Results

### Analysis of Stoichiometry of *S*. Typhi YfdX by SEC-MALS

**FIGURE 1:**
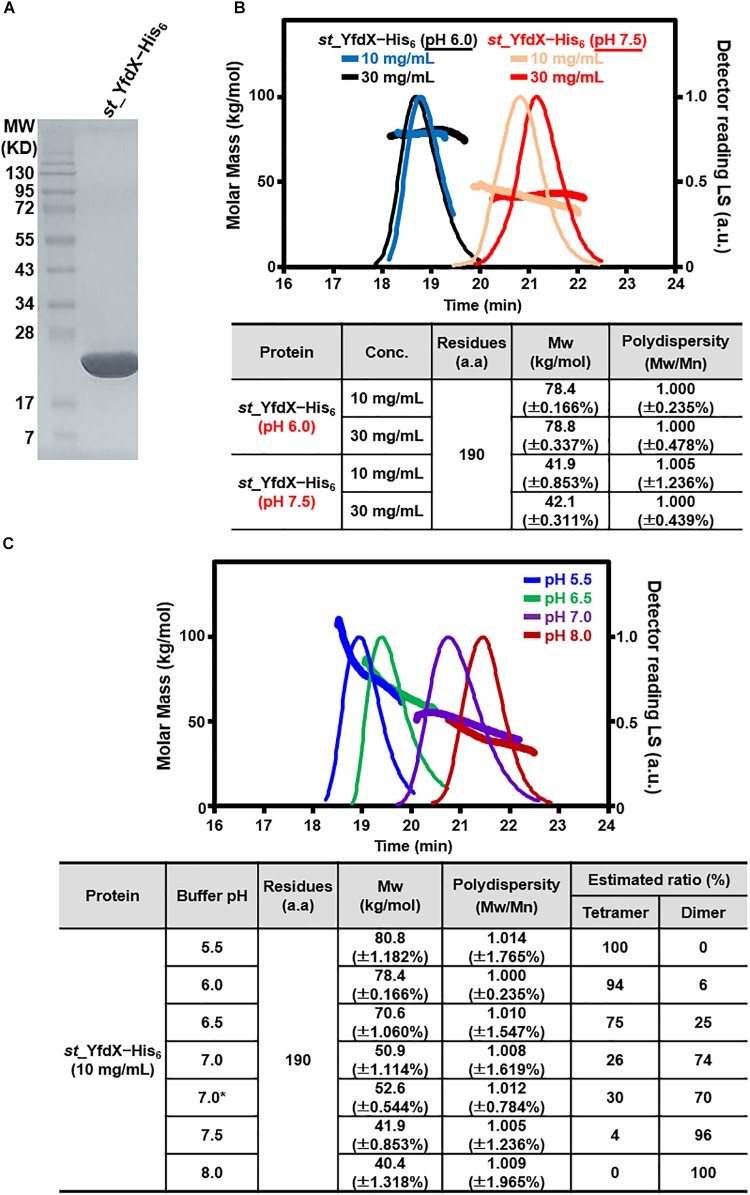
Preparation and SEC-MALS analysis of *st_*YfdX. **(A)** Purified *st_*YfdX–His_6_ (20 μg) was loaded onto a sodium dodecyl sulfate polyacrylamide gel electrophoresis system and visualized by Coomassie blue staining together with the size markers. **(B)** SEC-MALS analysis of wild-type *st_*YfdX–His_6_. (*Top*) Molar masses (in kg/mol) are plotted against the elution time (in min) from a size exclusion column. (*Bottom*) *st_*YfdX–His_6_ is in the form of a dimer at pH 7.5 and a tetramer at pH 6.0. Mw, weight-average molar mass; Mn, number-average molar mass. **(C)** *st_*YfdX is in pH-dependent dimer–tetramer equilibrium. (*Top*) SEC-MALS analysis was carried out under various pH conditions. (*Bottom*) Molecular weight of *st_*YfdX–His_6_ measured at the indicated condition are listed. The asterisk indicates the buffer condition (30 mM phosphate buffer pH 7.0 and 150 mM NaCl) used in the previous report (Saha et al., 2016b).

The recombinant *S*. Typhi YfdX protein containing residues 10–186 from its total 199 amino acids, referred to as *st_*YfdX in this manuscript, was overexpressed in *E. coli* and purified on a Ni–NTA column and a size-exclusion chromatography column, equilibrated with a final buffer consisting of 20 mM Tris-HCl (pH 7.5), 200 mM NaCl, and 1 mM DTT (Figure 1A). To verify the stoichiometry of *st_*YfdX by means of the recombinant protein, we carried out SEC-MALS experiments, which is a useful tool for elucidating the molecular weight and stoichiometry of macromolecules in solution (Sahin and Roberts, 2012). Unexpectedly, the molecular weight of wild-type *st_*YfdX, which was concentrated to 10 mg/mL, was determined to be ∼42 kDa, suggesting that this protein exists as a dimer in solution, neither a trimer nor a tetramer, under this condition (Figure 1B). Increasing the concentration of the protein sample to 30 mg/mL did not alter its stoichiometry (Figure 1B). Based on the presence of the zinc ion coordination at the center of the *K. pneumoniae* YfdX (referred to as *kp_*YfdX) tetrameric structure (not shown), we suspected that a similar coordination might be necessary for the formation of the high-order oligomeric structure of *st_*YfdX. Therefore, the recombinant *st_*YfdX protein was dialyzed against a buffer composed of 20 mM Bis-Tris-HCl (pH 6.0), 200 mM NaCl, and 1 mM DTT, with the intention of lowering pH to prevent zinc precipitation (Sayilgan et al., 2010; Park et al., 2015). Prior to zinc treatment, SEC-MALS experiments were conducted with the prepared protein sample, to use the data as a control. Surprisingly, we found that the molecular weights of the *st_*YfdX samples concentrated to 10 and 30 mg/mL, respectively, are ∼78–79 kDa (Figure 1B), and thus it was shown that *st_*YfdX forms a tetramer at pH 6.0, even without being treated with zinc chloride. The molecular weight of *st_*YfdX was then further analyzed by SEC-MALS at various pH conditions ranging from 5.5 to 8.0 at 0.5 intervals. As described in Figure 1C, the molar mass of *st_*YfdX was shifted from 80.8 to 40.4 kDa, indicating that *st_*YfdX exists in a state of dynamic equilibrium between tetramer and dimer whose ratio is varied by pH. We also performed SEC-MALS on the recombinant *kp_*YfdX protein for comparison. As shown in Supplementary Figure S1, *kp_*YfdX exists as a tetramer at pH 6.0 and 7.5 but as a dimer at pH 10.0, showing that *kp_*YfdX also undergoes a pH-dependent transition between dimeric and tetrameric forms but the pH range is different from that of *st_*YfdX. Collectively, these data indicate that the recombinant *st_*YfdX protein can form a dimer or tetramer in solution, and this process is affected by pH of the solution.

### Overall Structural Analysis of *st*_YfdX

**FIGURE 2:**
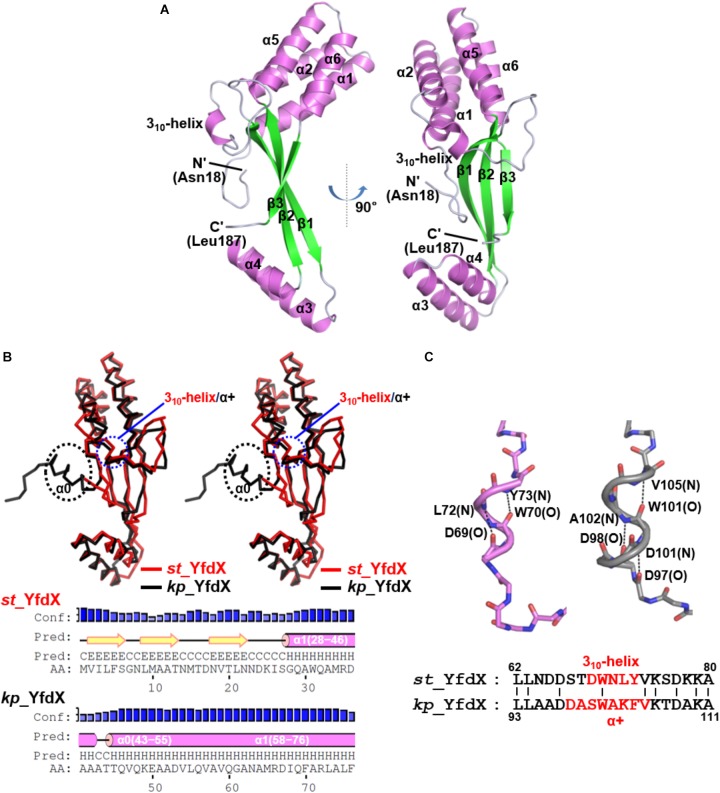
Crystal structure of the *st_*YfdX monomer. **(A)** Two views of the *st_*YfdX monomeric structure are presented as ribbon drawings with the labeled secondary structures. **(B)** (*Top*) Stereo views of superimposed *st_*YfdX (red) and *kp*_YfdX (black) monomers shown as C_α_ traces. Remarkable differences at the secondary structural level between the two molecules are indicated. (*Bottom*) Secondary structure prediction of the amino- terminal region of the two proteins, which was obtained from the PSI-PRED server (http://bioinf.cs.ucl.ac.uk/psipred/). **(C)** (*Top*) Structural comparison of the 3_10_-helix of *st_*YfdX (violet) and α+ of *kp*_YfdX (gray). Dashed lines highlight helical hydrogen bonding patterns with labels of the involved main chain amide or carbonyl groups. (*Bottom*) Sequence alignment. The residues constituting 3_10_-helix or α+ helices are highlighted in red.

Because structural information about *st_*YfdX was clearly indispensable for the corroboration of its stoichiometric properties, we attempted to elucidate its crystal structure. The purified recombinant protein was subjected to crystallization trials under 576 crystallization conditions. Rectangular crystals (space group *F*222) diffracted to the resolution of 1.4 Å were obtained in a crystallization condition with pH 4.6 (Crystal I; Tables 1, 2), implying that this crystal might consist of the *st_*YfdX tetramer. Its asymmetric unit contains a single *st_*YfdX molecule that is composed of six α-helices, one 3_10_-helix, and three β-strands (Figure 2A). These units are arranged to form three subdomains: a four-helical bundle (including α1, α2, α5, and α6), an antiparallel β-sheet (including β1–β3), and a two-helical bundle (including α3 and α4; Figure 2A). One 3_10_-helix is located in the middle of the α2–β1 loop (Figure 2A). The *st_*YfdX monomer overlaps well with the *kp_*YfdX monomer when superimposed, with a root mean square deviation of 1.27 Å over 154 aligned residues out of a total of 170 residues (Figure 2B, top), which is consistent with high sequence homology between them (35% identity and 52% similarity; Supplementary Figure S2). One noticeable discrepancy is the presence of an additional α-helix (referred to as α0) ahead of α1 of *kp_*YfdX, which is not shown in *st_*YfdX (Figure 2B). The PSI-PRED server that accurately predicted the presence of helical portion at that position in *kp_*YfdX suggested that residues 1–17 of *st_*YfdX, including residues 1–9 (not contained in the construct used for crystallization) and residues 10–17 (not visible in the crystal structure) might not form the α-helical structure (Figure 2B), supporting that *st_*YfdX does not contain α0. Another difference at the secondary structural level is that although the short helix of *st_*YfdX next to α2 is a 3_10_-helix containing i→i+3 hydrogen bonding, the corresponding helix of *kp_*YfdX is a canonical α-helix (referred to as α+) containing i→i+4 hydrogen bonding (Figure 2C). We noted that minor structural discrepancies between the two proteins are also evident in the 3_10_-helix–β1 and β1–α3 loops (Supplementary Figures 3A,B).

### Structural Analysis of the Oligomeric Form of *st_*YfdX

**FIGURE 3:**
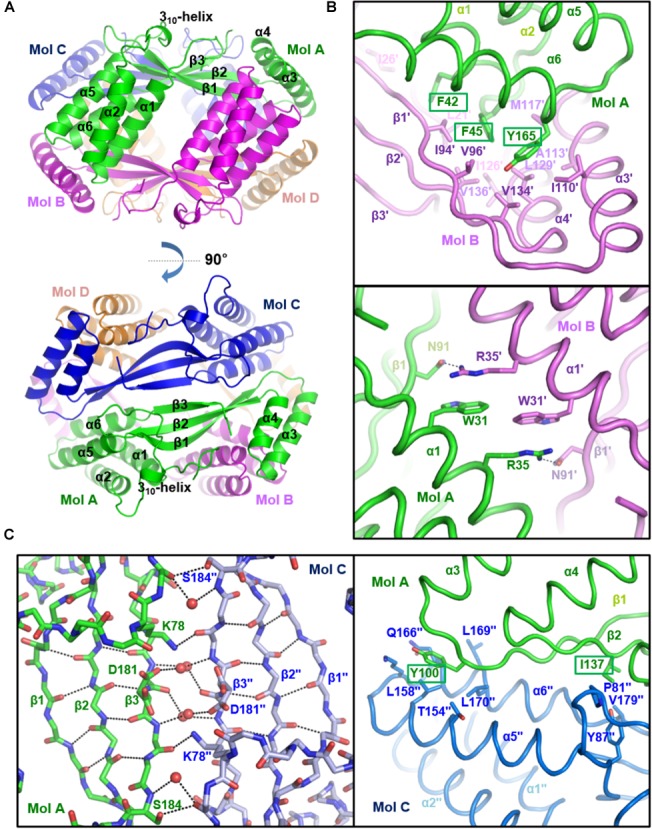
Crystal structure of *st_*YfdX tetramer. **(A)** Two views of the *st_*YfdX tetrameric structure are presented as ribbon models. For clarity, only the secondary structures of Mol A are labeled. **(B)** Intermolecular interaction between Mol A (green) and Mol B (violet) of *st_*YfdX. Shown as stick representation and labeled are *st_*YfdX residues participating in the oligomeric assembly. Three bulky aromatic residues (Phe42, Phe45, and Tyr165) centered at the hydrophobic-interacting interface are marked with rectangles (*top*). Hydrogen bonds between Arg35 from one molecule and Asn91 form the opposite molecule are presented as dotted lines (*bottom*). **(C)** Intermolecular interaction between Mol A (green) and Mol C (blue) of *st_*YfdX. (*Left*) Shown as stick representation are the main chain atoms of three-stranded β-sheets from the two molecules and the side chain atoms of Lys78, Asp181, and Ser184. Water molecules mediating intermolecular hydrogen bonds are presented as red circles. Intra- and inter-molecular hydrogen bonds are indicated by dotted lines. (*Right*) Shown as a stick model and labeled are residues participating in intermolecular hydrophobic interactions. Two hydrophobic residues (Tyr100 and Ile137) that are presumed to play a key role in the Mol A–Mol C binding interaction are marked with rectangles.

To verify the oligomeric state of *st_*YfdX, the intermolecular interactions and assembly were analyzed among *st_*YfdX molecules in crystals. Of note, we figured out that a tetramer-shaped oligomeric form of *st_*YfdX is shown in our crystal lattice (Figure 3A), which can be fairly well-matched to that of *kp_*YfdX (Supplementary Figure S4). In contrast, we could not find any crystallographic evidence for the formation of a *st_*YfdX trimer in our structure. We next structurally analyzed the intermolecular binding between *st_*YfdX monomers in the tetrameric form. The two *st_*YfdX molecules designated as Mol A and Mol B in Figure 3 interact with each other mainly through very tight hydrophobic contacts. These contacts are mediated by bulky aromatic hydrophobic residues (Phe42, Phe45, and Tyr165) at the tip of a four-helical bundle (especially α1 and α6) of one monomer and a number of hydrophobic residues (Leu21′, Ile26′, Ile94′, Val96′, Ile110′, Ala113′, Met117′, Ile126′, Leu129′, Val134′, and Val136′) in the hydrophobic concave region consisting of a two-helical bundle (α3 and α4) and at the tip of a three-stranded β-sheet of the other monomer (Figure 3B, top). Atomic analysis further revealed 85 intermolecular carbon–carbon contacts (<4.5 Å) in the MolA–MolB binding interface, with more than half being provided by the three aromatic residues Phe42, Phe45, and Tyr165 (51 C–C contacts; 60%). This hydrophobic interaction is well-conserved in the structure of the *kp_*YfdX tetramer (Supplementary Figure S3C, top) as well, because the contact-involved residues are mostly conserved among YfdX proteins (Supplementary Figure S2; marked with asterisks). It should be noted that residues such as Trp31, Arg35, and Asn91 from the two *st_*YfdX monomers also reinforce the intermolecular interaction by providing hydrophobic interaction between two tryptophan residues and hydrocarbon portion of two arginine residues and hydrogen bonds between Arg35 and Asn91 (Figure 3B, bottom). Similar but slightly different hydrophobic interactions and hydrogen bonds contribute to the intermolecular interaction between *kp_*YfdX molecules (Supplementary Figure S3C, bottom).

In the case of two *st_*YfdX molecules designated as Mol A and Mol C in Figure 3, at a glance, β-strands from the two monomers appear to form a continuous six-stranded β-sheet. However, we found that no typical hydrogen bond at less than 3.5 Å is present between two facing β-strands (β3 and β3^′′^) of the two *st_*YfdX monomers (Figure 3C, left), indicating that the six β-strands are discontinuous. Instead, the intermolecular association between the two three-stranded β-sheets is maintained by several side chains- and water molecules-mediated hydrogen bonds (Figure 3C, left). In the *kp_*YfdX crystal structure, the corresponding intermolecular interaction between two β-strands was reinforced by zinc ion coordination, in which two oxygen atoms from side chain carboxylates and four oxygen atoms from water molecules are involved (Supplementary Figure S3D, right). However, such a coordination is absent in the *st_*YfdX structure (Supplementary Figure S3D, left). Hydrophobic interactions provide additional force for the complex formation between Mol A and Mol C, which are mediated by Tyr100 and Ile137 from one molecule and Pro81^′′^, Tyr87^′′^, Leu158^′′^, Leu170^′′^, Val179^′′^, and the hydrocarbon part of Thr154^′′^, Lys163^′′^, and Gln166^′′^ from the opposite molecule (Figure 3C, right). Twenty-eight carbon–carbon contacts within 4.5 Å exist in the MolA–MolC binding interface, which is one third of those in the MolA–MolB binding interface. Similar hydrophobic interactions were also observed in the *kp_*YfdX structure (Supplementary Figure S3E), which are however a bit different from those in the *st_*YfdX structure along with the structural variation of β1–α3 loops (Supplementary Figure S3B). We found that Tyr100 and Ile137 of *st_*YfdX are conserved in *kp_*YfdX as Tyr131 and Ile168 (Supplementary Figure S2; marked with triangles), whereas their associating residues are relatively diverse in sequences between the two proteins. Overall, the interaction between Mol A and Mol B mainly depends on tight hydrophobic interaction and appears to be tight and dense (Figure 3B), whereas that between Mol A and Mol C is maintained by the combination of hydrogen bonds and loose hydrophobic interaction but seems to be relatively weak and detachable (Figure 3C).

### SEC-MALS and SAXS Exploration of *st*_YfdX

**FIGURE 4:**
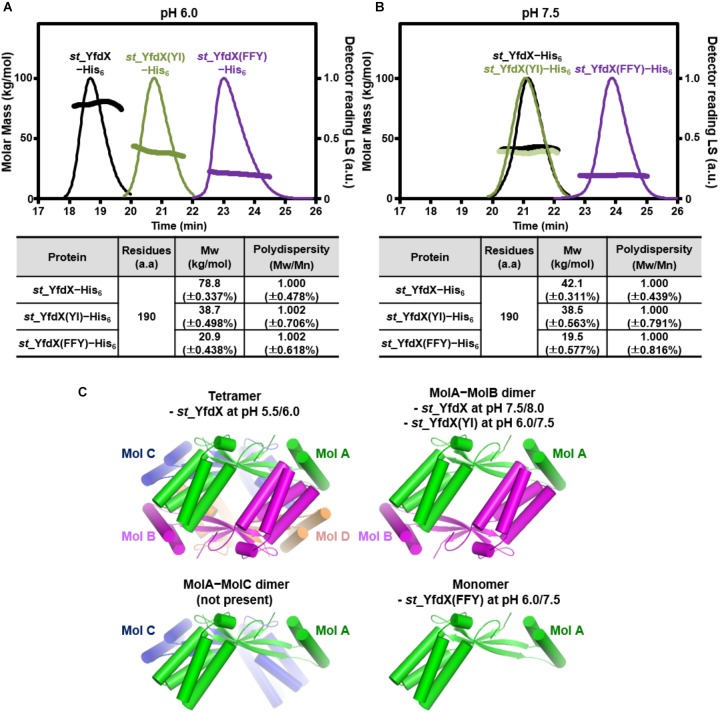
SEC-MALS analysis of mutant *st_*YfdX. **(A,B)** Molecular weights of the *st_*YfdX(FFY) and *st_*YfdX(YI) mutants were analyzed and compared with each other and with those of the wild-type. All the experiments were conducted on protein samples prepared at 30 mg/mL. **(C)** Structural diagrams of *st_*YfdX proteins according to the crystal structure and SEC-MALS experiments.

Whether the intermolecular interactions shown in the *st_*YfdX crystal structure indeed exist in solution was the next issue to be elucidated. Hence, we prepared two mutant *st_*YfdX proteins: *st_*YfdX(FFY), in which three core residues (Phe42, Phe45, and Tyr165; see Figure 3B, top) critical for the Mol A–Mol B interaction were substituted with alanine, and *st_*YfdX(YI), in which two hydrophobic residues (Tyr100 and Ile137; see Figure 3C, right) involved in the Mol A–Mol C interaction were mutated to alanine. These two proteins were expressed, purified, and finally equilibrated in two different solutions buffered with pH 6.0 Bis-Tris-HCl and pH 7.5 Tris-HCl, respectively, as was the wild-type protein. Introduction of such mutations did not affect *st_*YfdX protein folding, which was confirmed by CD spectroscopic analysis using wild-type and two mutant proteins (Supplementary Figure S5). SEC-MALS analysis was then performed on each protein sample, and the results were compared with one another and with those of wild-type. First, at both pH 6.0 and pH 7.5, the molecular weight of *st_*YfdX(YI) was ∼39 kDa (Figures 4A,B), indicating that structure-based alanine substitution of Tyr100 and Ile137 indeed abrogates the MolA–MolC interaction and therefore this mutant should exist as the MolA–MolB dimer (Figure 4C, right top). Next, the molecular weight of *st_*YfdX(FFY) was measured and found to be 20–21 kDa (Figure 4A) at both pH levels, showing that *st_*YfdX(FFY) exists in a monomeric form (Figure 4C, right bottom) but not as the MolA–MolC dimer (Figure 4C, left bottom). These data strongly indicate that when the very tight hydrophobic interaction-based MolA–MolB binding interface was impaired by the alanine substitution of Phe42, Phe45, and Tyr165, the relatively weak MolA–MolC interaction could not sustain the dimeric interface and thus it is in the monomeric form. As presented in Figure 1B, wild-type *st_*YfdX forms a tetramer at pH 5.5/6.0 but exists as a dimer at pH 7.5/8.0. We assume that the alteration of proton concentration might affect the formation of hydrogen bonds maintaining the MolA–MolC binding interface. The relatively weak MolA–MolC interaction is presumed not to be sustained at high pH conditions, and thus *st_*YfdX exists as the MolA–MolB dimer in that condition.

**FIGURE 5:**
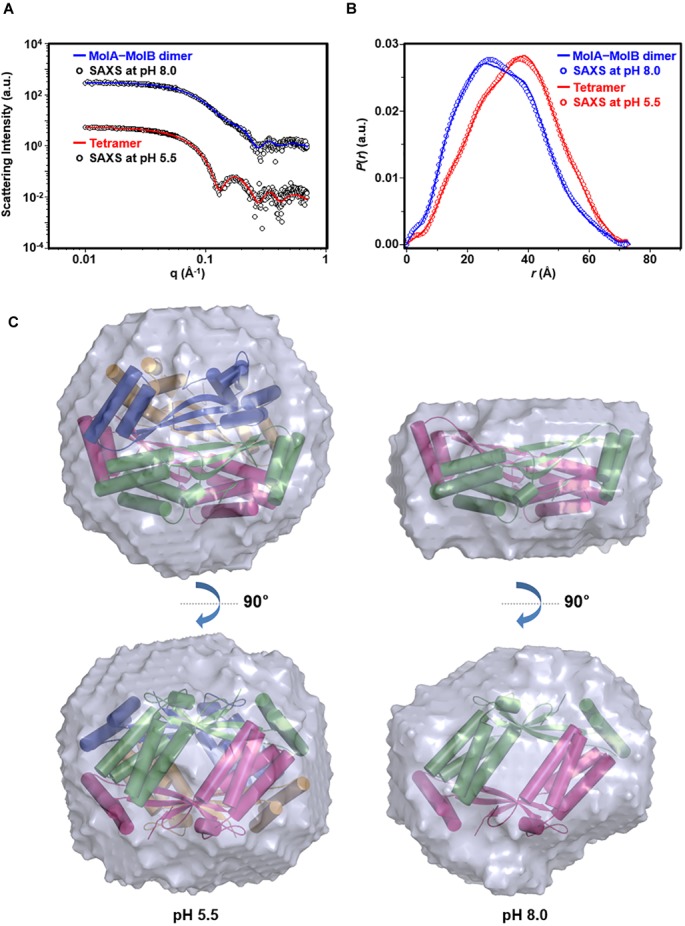
SAXS analysis of *st_*YfdX. **(A)** X-ray scattering profiles in solution measured at 4°C. Circles indicate experimental data, whereas solid lines represent the theoretical SAXS curve calculated from the tetrameric (χ^2^ = 0.583) and dimeric (χ^2^ = 0.483) models shown in Figure 4C using the program CRYSOL. For clarity, each plot is shifted along the log *I*(*q*) axis. **(B)** Pair distance distribution *p*(*r*) functions in aqueous solution that were acquired by analyzing the experimental SAXS data using the program GNOM. Areas under curves were normalized to equal areas for ease of comparison. **(C)** The tetrameric (left) and dimeric (right) models of *st_*YfdX were docked into the reconstructed SAXS envelopes that were reconstructed as described in detail in Section “Materials and Methods.”

To further corroborate the pH-dependent conversion of *st_*YfdX between the two forms, SAXS data were collected using two protein samples equilibrated at pH 5.5 and 8.0, respectively (Table 3). We first confirmed that the calculated scattering intensity curves and distance distribution function *P*(*r*) of the tetrameric and the MolA–MolB dimeric structures were nicely matched with the experimental observations (Figures 5A,B). Subsequently, the molecular envelope of the samples was derived from SAXS analysis. The envelope of *st_*YfdX at pH 5.5 was globular-shaped, in which the tetrameric crystal structure of *st_*YfdX could be successfully incorporated (Figure 5C, left). In contrast, that of *st_*YfdX at pH 8.0 was revealed to have a hemisphere-like shape that fitted well the MolA–MolB dimer (Figure 5C, right). Collectively, SEC-MALS and SAXS data demonstrate that *st_*YfdX switches between two different forms in a pH-dependent manner in the solution, whose conformation could be inferred from the crystal structure.

**Table 3:** Structural parameters obtained from the SAXS data of *st*_YfdX proteins in solution.

Model/sample	*R*_g,G_^c^ (Å)	*R*_g,p(r)_^d^ (Å)	*D*_max_^e^ (Å)	*MM*_calculated_^f^ (kDa)	*MM*_SAX_^g^ (kDa)	*MM*_MALS_^h^ (kDa)
Tetrameric model^a^	27.39 ± 0.04	26.91 ± 0.04	73.4	82.4	–	–
MolA-MolB model^a^	23.95 ± 0.01	23.86 ± 0.14	73.5	41.2	–	–
*st*_YfdX-His_6_ at pH 5.5^b^	27.54 ± 0.21	26.90 ± 0.09	72.5	–	80.4	80.8
*st*_YfdX-His_6_ at pH 8.0^b^	23.82 ± 0.21	23.84 ± 0.12	72.1	–	38.2	40.4

*^a^Models obtained from the st_YfdX structure determined using the crystal I. ^b^Samples subjected to SAXS. ^c^R_g_,_G_ (radius of gyration) was obtained from the scattering data by the Guinier analysis. ^d^R_g,p(r)_ (radius of gyration) was obtained from the p(r) function by the program GNOM. ^e^D_max_ (maximum dimension) was obtained from the p(r) function by the program GNOM. ^f^MM_calculated_ (molecular mass) was obtained from the amino acid sequence of protein. ^g^MM_SAXS_ (molecular mass) was obtained from the SAXS experiments using BSA as a standard. ^h^MM_MALS_ (molecular mass) was obtained from the MALS experiments using BSA as a standard (see Figure 1C).*

### Crystallization and Structure Determination of ZnCl_2_-Treated *st_*YfdX

As mentioned above, the pH-sensitive stoichiometric alteration of *st_*YfdX was detected during examination of the possibility of zinc coordination-mediated oligomerization. Even though such a zinc coordination was not shown in our crystal structure (Supplementary Figure S3D), we did not exclude such a possibility completely, and thus attempted crystallization of the *st_*YfdX protein sample equilibrated with the final buffer consisting of 20 mM Bis-Tris-HCl (pH 6.0), 200 mM NaCl, 1 mM DTT, and 1 mM ZnCl_2_. Two forms of crystals were obtained that were diffracted to high resolution: rectangular crystals diffracted to 1.5 Å (space group *F*222; crystal II) under the same crystallization condition as that of crystal I, and board-shaped crystals diffracted to 2.3 Å (space group *P*222; crystal III) under the crystallization condition at pH 5.0 (see Table 1). The asymmetric units of crystals II and III contain one and eight *st_*YfdX molecules, respectively. We once again determined the structure of *st_*YfdX using these crystals (Table 2). The *st_*YfdX structures determined via crystals I–III were confirmed to be almost identical to each other; not only because the *st_*YfdX monomeric structures from crystals II and III were superimposed on each other and on that from crystal I with root mean square deviations in the range of 0.08–0.77 Å over ∼170 aligned residues, but also because tetramer-shaped oligomeric forms of *st_*YfdX were detected in the crystal lattices of both crystals II and III, as was the case for crystal I (Supplementary Figure S4). These data support that *st_*YfdX can form a tetramer. Nevertheless, oligomerization-contributing zinc coordination demonstrated in the *kp_*YfdX structure (Supplementary Figure S3B, right) was not found in all our crystal structures of *st_*YfdX (not shown), indicating that the zinc coordination is not necessary for formation of the tetramer of this protein.

Next, to understand the biological function of *st_*YfdX, a search for homologous structures on the Dali server (Holm and Sander, 1993) was carried out with the monomeric and tetrameric structures of *st_*YfdX. No homologous protein was identified in this search except for *kp_*YfdX (Z-score 20.1): a number of proteins containing a helical bundle were listed including *Nitrosomonas europaea* small metal-binding protein (Z-score 9.9) and *E. coli* cytochrome *b*_562_ (Z-score 8.5), but their structures overlapped with the *st_*YfdX structure only partially (Supplementary Figure S6), indicating that YfdX adopts a novel protein fold that has not been identified yet.

### YfdX Modulates Antibiotic Susceptibility in *Salmonella*

**FIGURE 6:**
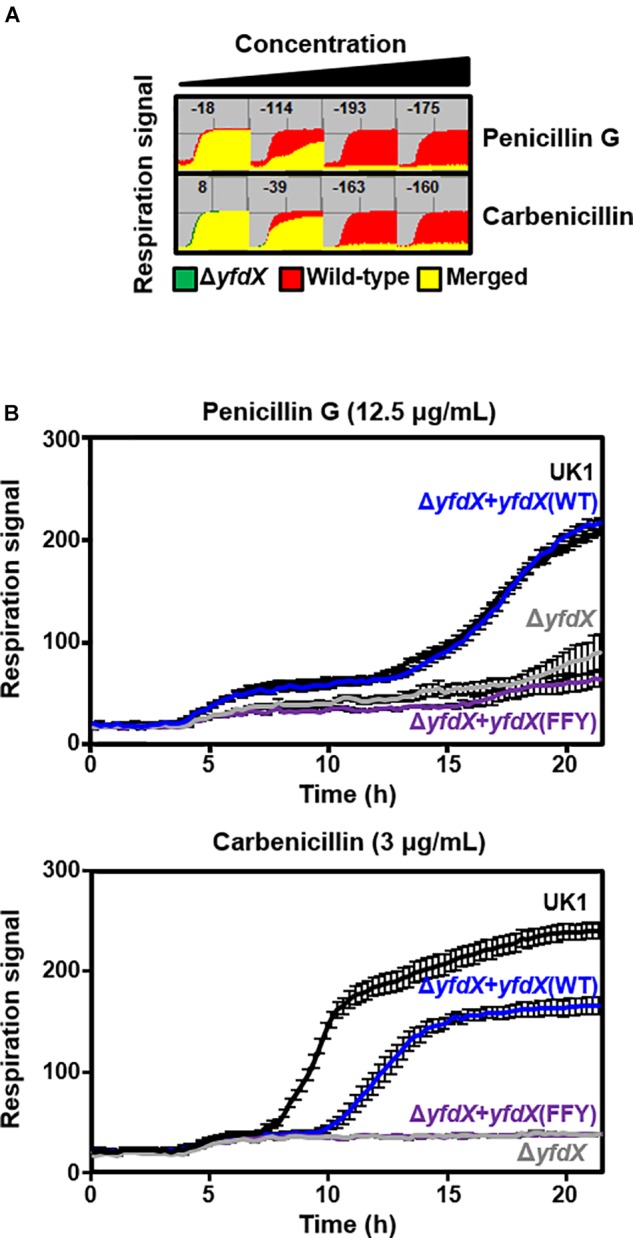
Functionality of YfdX in antibiotic tolerance. **(A)** Phenotype arrays. Deficiency of YfdX enhanced susceptibility of *S*. Typhimurium to the indicated antibiotics. **(B)** Respiration of the *S*. Typhimurium UK-1 strains from 0 to 25 h was measured in the presence of each antibiotic at the indicated concentration.

Previous reports indicate that protein expression of *E. coli* YfdX is under control of EvgA, the response regulator factor that is intimately associated with the bacterial response to antibiotic stress (Nishino and Yamaguchi, 2001, 2002; Masuda and Church, 2002; Nishino et al., 2003). Even though the homolog of EvgA is absent in the genome of *S.* Typhi, we assumed that YfdX might also play a role in the response to antibiotics. Therefore, we analyzed the functional and physiological effects of *yfdX* gene expression in the *S*. Typhimurium UK-1 strain, whose YfdX (referred to as *stm*_YfdX in this manuscript) shares 98.0% identity (195 of 199 amino acids) with *st*_YfdX. First, the cellular expression level of *stm*_YfdX was examined in a time-dependent manner. As illustrated in Supplementary Figure S7, the transcription level of *stm*_YfdX was proportionally increased every 4 h and was higher at 12 h than at 4 or 8 h, indicating that cellular *stm*_YfdX was expressed in the exponential and, particularly, stationary phases. Next, phenotype microarrays were conducted by measuring the bacterial respiration rate to compare physiological changes upon antibiotic treatment between *S*. Typhimurium UK-1 wild-type and *yfdX*-deficient (Δ*yfdX*) strains. We found that the absence of YfdX makes the bacterium more susceptible to two antibiotics that are known to block cell wall synthesis, penicillin G and carbenicillin (Figure 6A). Figure 6B corroborates that the growth of the Δ*yfdX* strain was remarkably restrained in the presence of each of the two antibiotics at the concentration where the wild-type strain grows well. Moreover, the growth of the Δ*yfdX* strain was completely (when treated with penicillin G) or considerably (when treated with carbenicillin) recovered by chromosomal complementation of the wild-type *S*. Typhi *yfdX* gene but not by that of the *yfdX* gene containing the FFY mutation (Figure 6B). These data collectively indicate that *Salmonella* YfdX performs significant functions in basal tolerance of this pathogenic bacterium against β-lactam antibiotics, and that its homooligomerization might be necessary for the functionality of this protein.

### YfdX Deficiency in *Salmonella* Results in Promoted Bacterial Virulence

**FIGURE 7:**
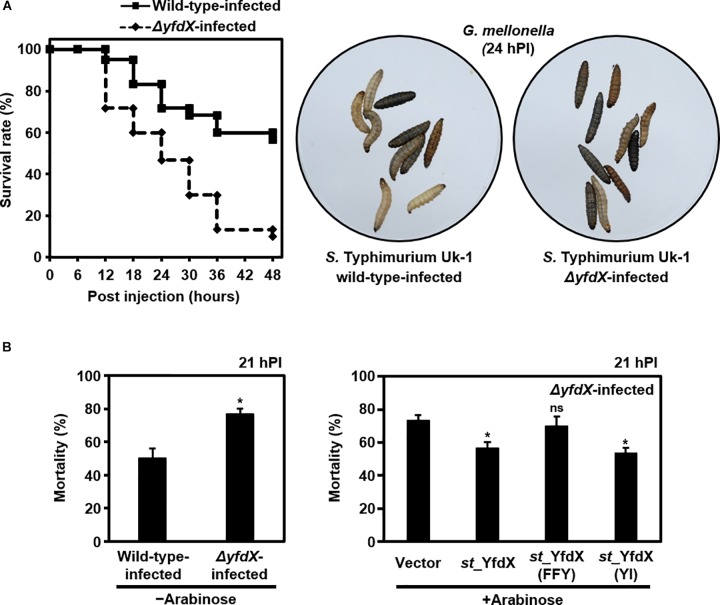
YfdX reduces bacterial virulence. **(A)** *yfdX* deficiency enhanced *Salmonella* virulence to *Galleria mellonella* larvae. (*Left*) The survival rates of larvae infected by the *S*. Typhimurium UK-1 wild-type or Δ*yfdX* strain were measured every 6 h and compared. (*Right*) Pictures of *Salmonella*-infected larvae 24 h post-infection. White ones are alive, whereas black ones are dead. **(B)** Complementation assay. (*Left*) Mortality rates of wild-type or Δ*yfdX* *Salmonella* strain-infected larvae are compared. ^∗^*P* < 0.05 (Student’s *t*-test). (*Right*) Comparison of mortality rates of Δ*yfdX* *Salmonella*-infected larvae with the arabinose-induced transient expression of an empty vector (control), *st*_YfdX, *st*_YfdX(YI), or *st*_YfdX(FFY). ns, not significant; ^∗^*P* < 0.05 versus transient expression of empty vector (Student’s *t*-test).

Given that various bacterial stress regulators are also known to be involved in bacterial virulence (Fang et al., 2016), we wondered whether *Salmonella* YfdX is able to modulate microbial virulence as well. A virulence screening system that is based on *G. mellonella* larvae as the *in vivo* model for *Salmonella* infection was employed to determine this issue. After injection of the *S*. Typhimurium UK-1 wild-type or Δ*yfdX* strain, the larval survival rate was recorded every 6 h for up to 48 h. Of note, the survival rate of larvae infected by the Δ*yfdX* strain (∼10% at 48 h) was significantly lower than that of larvae infected by the wild-type strain (∼60% at 48 h), indicating that *stm*_YfdX plays a role in attenuating bacterial virulence (Figure 7A). To confirm these results, we prepared *S*. Typhimurium UK-1 Δ*yfdX* strains in which YfdX-deficiency was complemented by arabinose-induced transient expression of wild-type *st*_YfdX or crystal structure-based mutant *st*_YfdX proteins including *st*_YfdX(FFY) and *st*_YfdX(YI) (see Figures 3, 4). We first reconfirmed that the Δ*yfdX* strain-infected larvae show remarkably higher mortality than that of the wild-type strain-infected larvae at 21 h post-injection (Figure 7B, left). Subsequent analysis revealed that the *Salmonella* virulence was relieved by the transient expression of wild-type *st*_YfdX or *st*_YfdX(YI), but not by that of *st*_YfdX(FFY). Because *st*_YfdX(YI) and *st*_YfdX(FFY) exist in a dimeric and monomeric form, respectively (see Figure 4), these results imply that the oligomeric form but not the monomeric form of *st*_YfdX is functional and necessary to suppress *Salmonella* virulence. We also confirmed similar effects of *st*_YfdX on the larval survival rate by means of the *S*. Typhimurium 14028S strain (Supplementary Figure S8).

## Discussion

### Analysis of the Oligomeric Status of YfdX

In this study, we performed X-ray crystallographic and scattering, biochemical, and physiological analyses of *Salmonella*-derived YfdX, whose characteristics and biological function remain obscure despite recent research (Saha et al., 2016a,b; Mondal et al., 2017). One of the issues raised by a study on *st_*YfdX is about its stoichiometry (Saha et al., 2016b): *st_*YfdX indeed forms a trimer unlike *kp*_YfdX that appears to form a tetramer despite their high sequence homology. SEC-MALS analysis and structure determination by X-ray crystallographic and scattering analyses were carried out to resolve this issue, which led to the unexpected identification of a pH-related stoichiometric switch of *st_*YfdX (Figures 1–5). Although both the tetrameric (at pH 7.5/8.0) and dimeric form (at pH 5.5/6.0) were confirmed by SEC-MALS (Figure 1) and SAXS (Figure 5), *st_*YfdX was found to be in the tetrameric form in all the three crystal structures, presumably because of the increased opportunity of contacts in the crystal lattice (Figure 3 and Supplementary Figure S4). Nevertheless, the dimeric structure of *st_*YfdX could be inferred from the combination of a structure-based mutational study accompanied by SEC-MALS analysis. Among the two hypothetical dimers illustrated in Figure 4C, the MolA–MolB form, but not the MolA–MolC form, is presumed to exist in solution, for the following reasons. First, the intermolecular interactions between MolA and MolB are clearly tighter and denser than those between MolA and MolC (Figures 3B,C). Second, the MolA–MolB interaction-null *st_*YfdX(FFY) mutant was revealed to be a monomer, not a dimer (Figure 4B), implying that the MolA–MolC interaction is not strong enough to sustain the complex formation. We speculate that the tetrameric form of *st_*YfdX observed at pH < 6.0 might involve assembly of two MolA–MolB dimers. SEC-MALS analysis also showed a pH-dependent gradual shift of the calculated molecular mass of *st_*YfdX from pH 5.5 to 8.0, indicating that *st_*YfdX is in a state of dynamic equilibrium between tetramer and dimer (Figure 1C). At pH 7.0, the molecular weight of *st_*YfdX was calculated to be ∼51–53 kDa, which presumably explains the reason that *st_*YfdX was estimated to form a trimer in the previous report (Saha et al., 2016b). Given the SEC-MALS analysis together with the crystallographic and SAXS structural analysis collectively (Figures 1–5), we consider that *st_*YfdX undergoes a continuous structural switch between dimer and tetramer rather than exists as a trimer.

Determination of the stoichiometry of *st_*YfdX was followed by corollary issues concerning the biological importance of such oligomerization. Chromosomal knockout and complementation assay using the *S*. Typhimurium UK-1 strain demonstrated that the monomer-forming mutant YfdX is non-functional (see Figure 6). The *G. mellonella* larvae-infection assay showed that the genetic knockout of YfdX could be complemented by the transient expression of the wild-type or the dimer-forming mutant, but not by that of the monomer-promoting mutant (see Figure 7). These data collectively suggest that the oligomerization of *st_*YfdX might be inevitable for its effectiveness. It is notable that we currently do not rule out the probability that the tetrameric and dimeric forms function differently in other cellular processes in which s*t_*YfdX is involved.

### YfdX Acts a Critical Dual Function in Antibiotic Susceptibility and Virulence

Despite previous efforts examining the diverse biochemical properties of s*t_*YfdX (Saha et al., 2016a,b; Mondal et al., 2017), analysis of its functionality in live bacteria has been absent thus far, limiting our understanding of this protein. In this study, we demonstrated that YfdX is indeed involved in *Salmonella*’s tolerance to antibiotics, because its deficiency caused a remarkable increase in antibiotic susceptibility and impaired bacterial survival upon treatment with penicillin G and carbenicillin, which was compensated by chromosomal complementation with the wild-type *yfdX* but not with the monomer-promoting mutant *yfdX* (Figure 6). We also observed that the expression level of YfdX in *Salmonella* was higher in the stationary phase than that in the exponential phase (Supplementary Figure S7). Because tolerance to a variety of environmental stressors such as antibiotics and osmotic or acidic stress is vital for bacterial species, particularly in the stationary phase where bacterial growth is delayed or arrested, a number of bacterial proteins that are upregulated in this phase were found to be involved in a stress response and/or antibiotic tolerance (Hengge-Aronis, 1993; Battesti et al., 2011), which now should include YfdX. In addition, it was revealed for the first time that YfdX mitigates mortality of the *Salmonella* infection in our larval model (Figure 7), suggesting that YfdX is a negative regulator of bacterial virulence. Even though antibiotic resistance and bacterial virulence are different in their functionality and applicability, a number of biological processes have been reported to be shared by the two mechanisms, including biofilm formation (Patel, 2005), upregulated expression of an efflux pump (Barbosa and Levy, 2000), regulation of cell permeability (Tsai et al., 2011), and cell wall alteration (Moya et al., 2008). Moreover, in a variety of gram-negative pathogenic bacteria such as *Staphylococcus aureus* and *Streptococcus pneumoniae*, bacteria strains resistant to antimicrobial molecules including β-lactams and glycopeptides show attenuated virulence when they infect mice (Rieux et al., 2001; Rudkin et al., 2012) or *G. mellonella* (Peleg et al., 2009), implying an association between the two mechanisms at the molecular level. Yet, the precise molecular mechanism of YfdX’s action in the control of *Salmonella* antibiotic tolerance and bacterial virulence remains to be elucidated. One possibility is that YfdX may associate with membrane proteins and affect their function or localization, e.g., proteins involved in cell wall synthesis, as its deficiency led to the increased susceptibility to β-lactam antibiotics such as penicillin G and carbenicillin (Figure 6). Since a recent report shows simulated binding of *st*_YfdX to the outer-membrane protein STY3179, a *Salmonella* homolog of an adhesion and invasion locus protein from *Yersinia pestis* that interacts with the host extracellular matrix protein laminin (Yamashita et al., 2011), another possibility is that YfdX controls bacterial virulence by regulating host–pathogen interaction via binding to STY3179 or other unknown proteins. We suppose that future verification of the binding partner(s) of YfdX will be necessary to resolve this issue.

It has been reported that the EvgS-EvgA system controlling the expression of the *yfdXWUVE* operon in *E. coli* regulates the signaling pathways also associated with acid tolerance (Nishino and Yamaguchi, 2001, 2002; Masuda and Church, 2002; Nishino et al., 2003). We, therefore, raise a possibility that st_YfdX might participate not only in antibiotic susceptibility but also in acid tolerance of the bacterium or other processes such as pH homeostasis that is also implicated in *Salmonella* virulence (Rappl et al., 2003), in which its pH-dependent structural conversion could play some unknown role. However, at least under our assay conditions, the relation of st_YfdX with acid shock was shown to be unclear and inconsistent in the phenotype microarrays and in the minimum inhibitory concentration assays (unpublished data), and this issue remains to be resolved by future research as well.

## Conclusion

**FIGURE 8:**
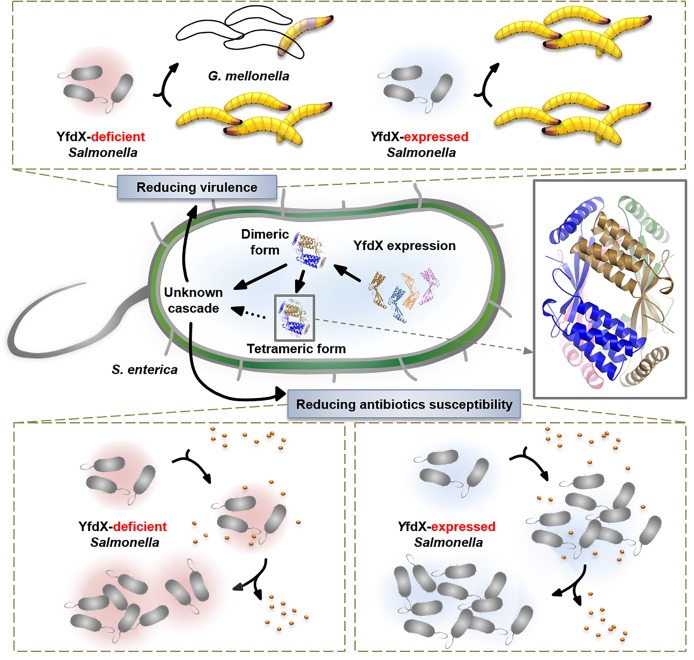
Overall view of the physiological functionality of YfdX.

We characterized various features of the *Salmonella*-derived YfdX protein, including the crystal structure that contains a novel fold, previously unknown stoichiometric features, proof of contribution to antibiotic response, and participation in the control of bacterial virulence (Figure 8). We believe that our biochemical and physiological findings will provide a rational basis for future research, augmented with the details of molecular mechanisms of the stress response in this pathogenic bacterium, in which much remains to be discovered.

## Author Contributions

BK, C-MR, and SJK conceived and designed the experiments. HSL, SL, J-SK, H-RL, and KSJ performed the experiments. HSL, SL, KSJ, BK, C-MR, and SJK analyzed the data. H-CS, M-SL, and C-HK contributed to reagents, materials, analysis tools. HSL, J-SK, BK, C-MR, and SJK wrote the manuscript.

## Conflict of Interest Statement

The authors declare that the research was conducted in the absence of any commercial or financial relationships that could be construed as a potential conflict of interest.

## Footnotes

1 The coordinates and the structure factors of the *st_*YfdX structures determined using crystals I, II, and III have been deposited in the PDB under the accession codes 6A02, 6A07, and 6A09, respectively.

## Abbreviations

CD: circular dichroism
DTT: dithiothreitol
MDR: multiple drug resistance
PDB: Protein Data Bank
SAXS: small-angle X-ray scattering
SEC-MALS: size exclusion chromatography-multiangle light scattering
