# Supplementary material for: Structural and Physiological Exploration of Salmonella Typhi YfdX Uncovers Its Dual Function in Bacterial Antibiotic Stress and Virulence
Source: Front Microbiol. 2019 Jan 14;9:3329. doi: 10.3389/fmicb.2018.03329 (PMC6339873; doi:10.3389/fmicb.2018.03329)
Supplement: Supplementary file 1 [file Data_sheet_1.docx]

**Supplementary Materials**

**Structural and physiological exploration of *Salmonella* Typhi YfdX uncovers its dual function in bacterial antibiotic stress and virulence**

Hye Seon Lee^1,2†^, Soohyun Lee^3†^, Jun‐Seob Kim^3^, Hae-Ran Lee^3^, Ho-Chul Shin^1^, Moo-Seung Lee^3^, Kyeong Sik Jin^4^, Cheol-Hee Kim^2^, Bonsu Ku^1*^, Choong-Min Ryu^3,5*^ and Seung Jun Kim^1,6*^

^1^Disease Target Structure Research Center, Korea Research Institute of Bioscience and Biotechnology, Daejeon 34141, Korea

^2^Department of Biology, Chungnam National University, Daejeon 34134, Korea

^3^Infectious Disease Research Center, Korea Research Institute of Bioscience and Biotechnology, Daejeon 34141, Korea

^4^Pohang Accelerator Laboratory, Pohang University of Science and Technology, Pohang, Kyungbuk 37673, Korea

^5^Department of Biotechnology, University of Science and Technology KRIBB School, Daejeon 34113, Korea

^6^Department of Bioscience, University of Science and Technology KRIBB School, Daejeon 34113, Korea

Running Title: Molecular characterization of *Salmonella* YfdX

Keywords: STY3178, YfdX, *Salmonella* Typhi, antibiotics susceptibility, virulence

*Correspondence: Bonsu Ku, [bku@kribb.re.kr](mailto:bku@kribb.re.kr); Choong-Min Ryu, [cmryu@kribb.re.kr](mailto:cmryu@kribb.re.kr); Seung Jun Kim, [ksj@kribb.re.kr](mailto:ksj@kribb.re.kr)

^†^These authors have contributed equally to this work

**
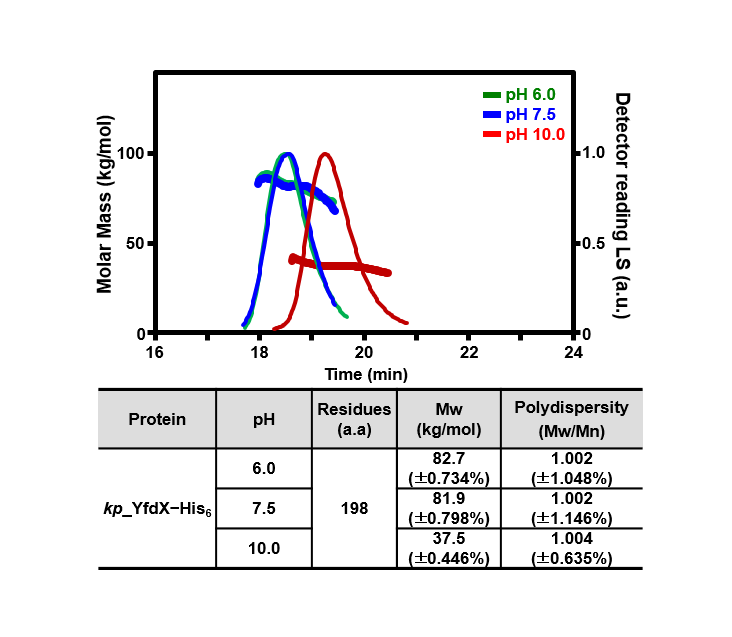
**

**Supplementary Figure S1. SEC-MALS analysis of *kp_*YfdX**

Molecular weight of *kp_*YfdX equilibrated with a 100 mM NaCl and 1 mM DTT-containing solution buffered by 20 mM Bis-Tris-HCl (for pH 6.0), Tris-HCl (for pH 7.5), or CAPS-NaOH (for pH 10.0) was analyzed by SEC-MALS.

**
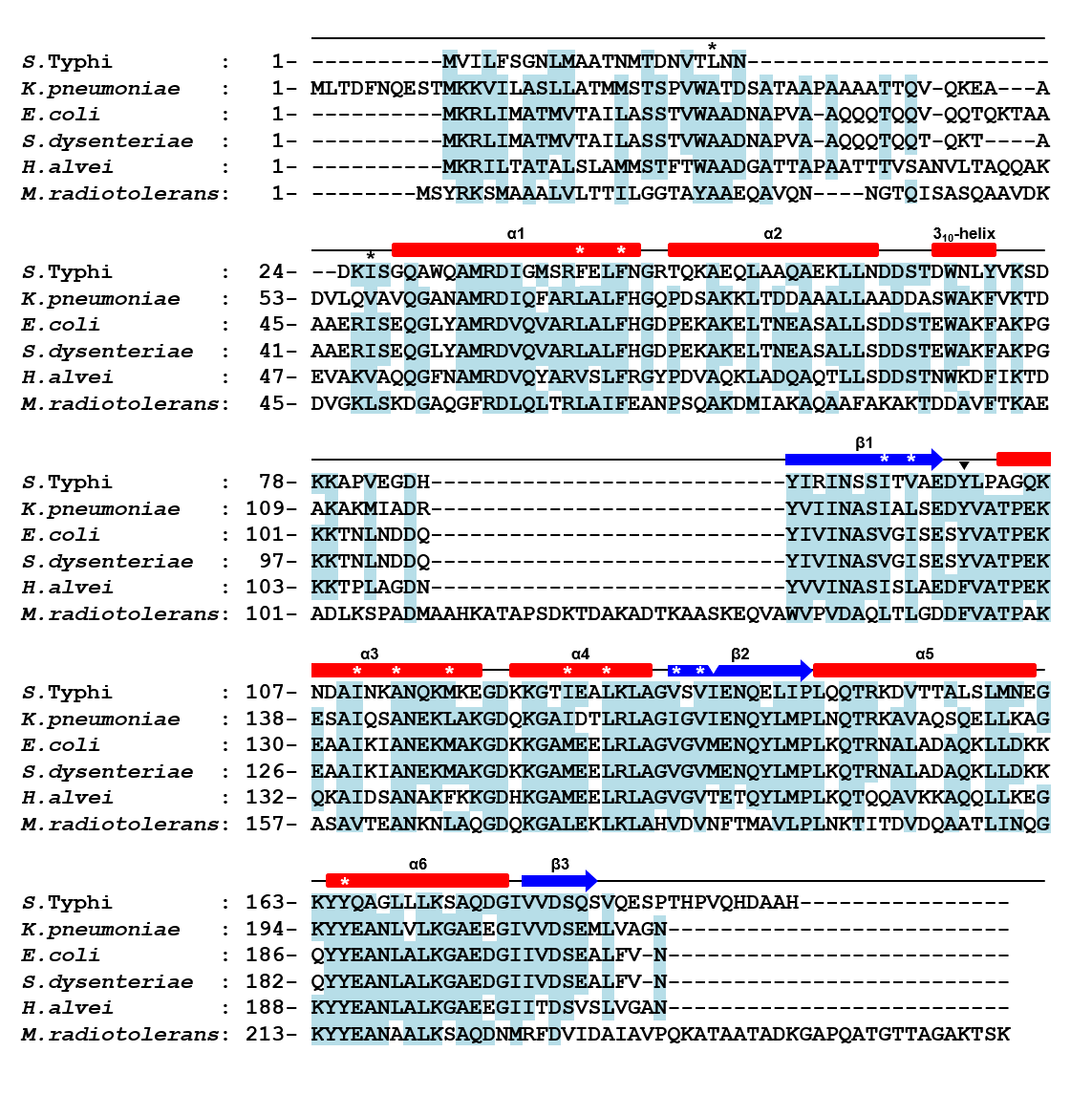
**

**Supplementary Figure S2. Sequence alignment**

The amino acid sequences of six different bacterial YfdX proteins are aligned. The secondary structures of *st_*YfdX are shown together. Conserved residues (sequence identity > 60%) are shaded in cyan. The key residues for the intermolecular hydrophobic interaction among *st_*YfdX molecules are indicated by asterisks (see Figure 3B, top) and triangles (see Figure 3C, right).

**
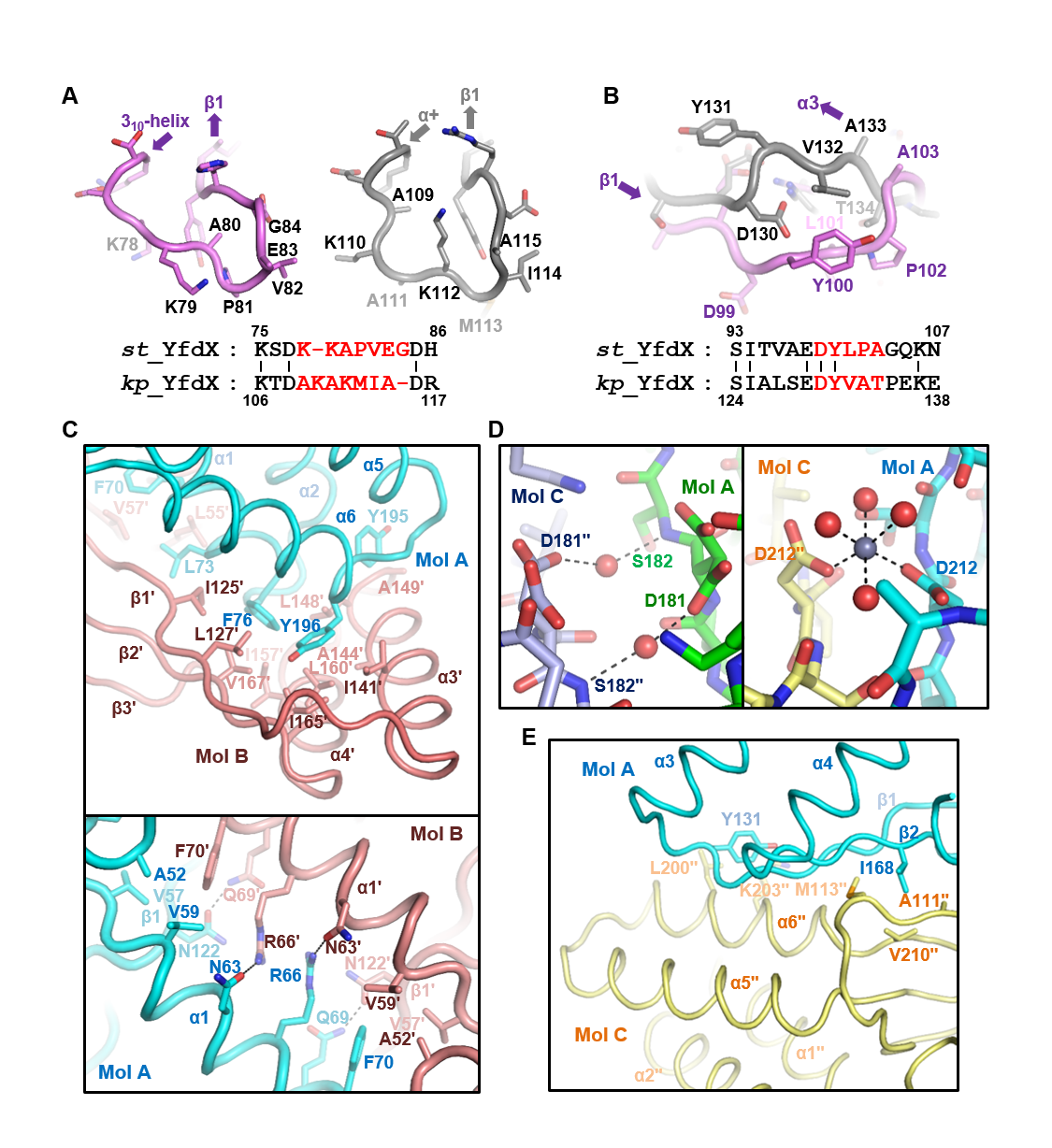
**

**Supplementary Figure S3. Structural comparison between *st_*YfdX and *kp_*YfdX**

(A−B) 3_10_-helix–β1 (*A*) and β1–α3 (*B*) loops of *st_*YfdX are structurally aligned with the corresponding loops of *kp*_YfdX (*top*). Sequence alignments are shown below, in which the compared residues are highlighted in red (*bottom*).

(C) Intermolecular interactions between Mol A (cyan) and Mol B (sepia) of *kp*_YfdX are shown at the same angle as those of *st_*YfdX shown in Figure 3B. Residues that are involved in the oligomeric assembly are presented as a stick model and labeled. Intermolecular hydrogen bonds are represented as dotted lines.

(D) Zinc ion coordination is shown in the oligomeric interface of *kp*_YfdX (*right*), but not in that of *st_*YfdX (*left*). Navy and red circles indicate zinc and water molecules, respectively. Water molecule-mediated hydrogen bonds and zinc ion coordination are shown as dotted lines.

(E) Intermolecular interactions between Mol A (cyan) and Mol C (yellow) of *kp*_YfdX are shown at the same angle as those of *st_*YfdX, as illustrated in Figure 3C (right). Residues involved in the oligomeric assembly are shown as stick representation and labeled.

**
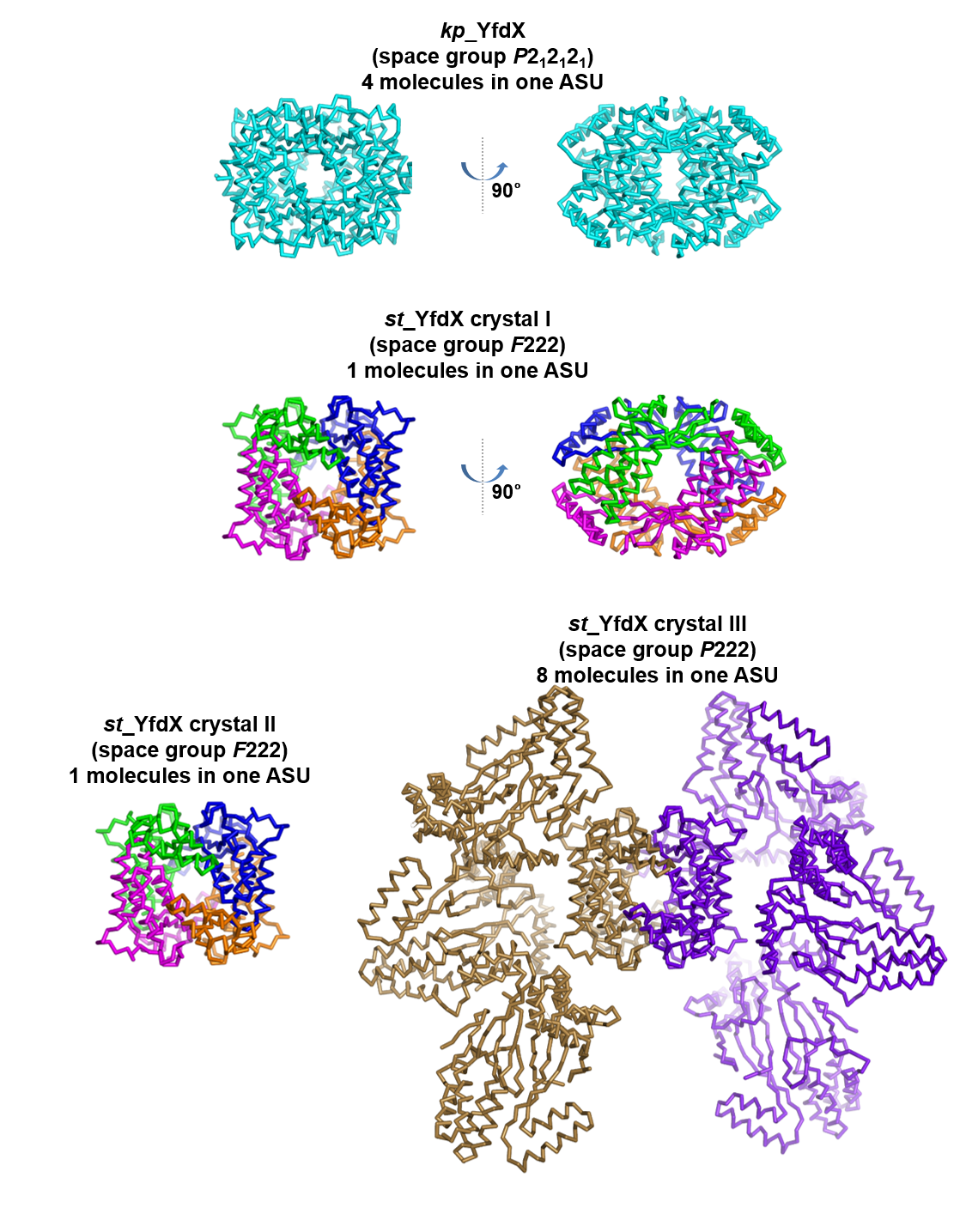
**

**Supplementary Figure S4. Assembly of *kp*_YfdX and *st_*YfdX molecules in crystals**

YfdX protein molecules are shown in C_α_ traces as assembled in crystals. In each structure, molecules in the single asymmetric unit have identical colors.

**
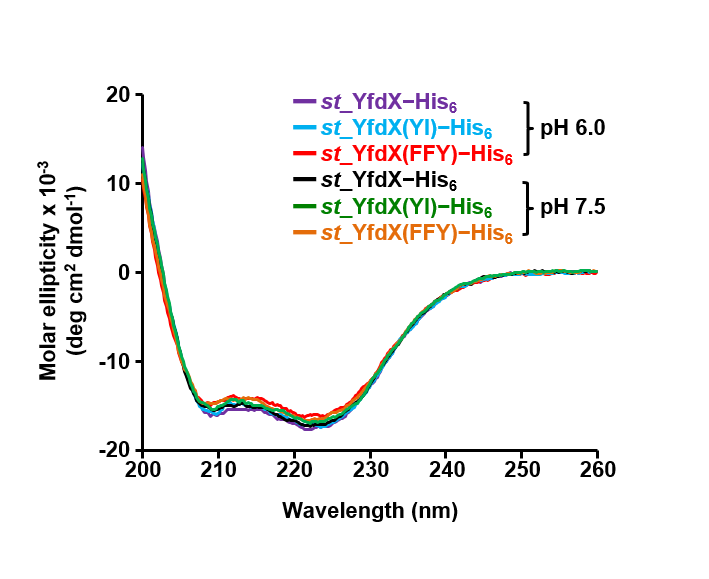
**

**Supplementary Figure S5. CD analysis of *st_*YfdX proteins**

Wild-type and two mutant *st*_YfdX proteins at pH 6.0 or pH 7.5 were subjected to CD spectroscopic analysis. The resulting molar ellipticity curves were nearly the same among the six samples.


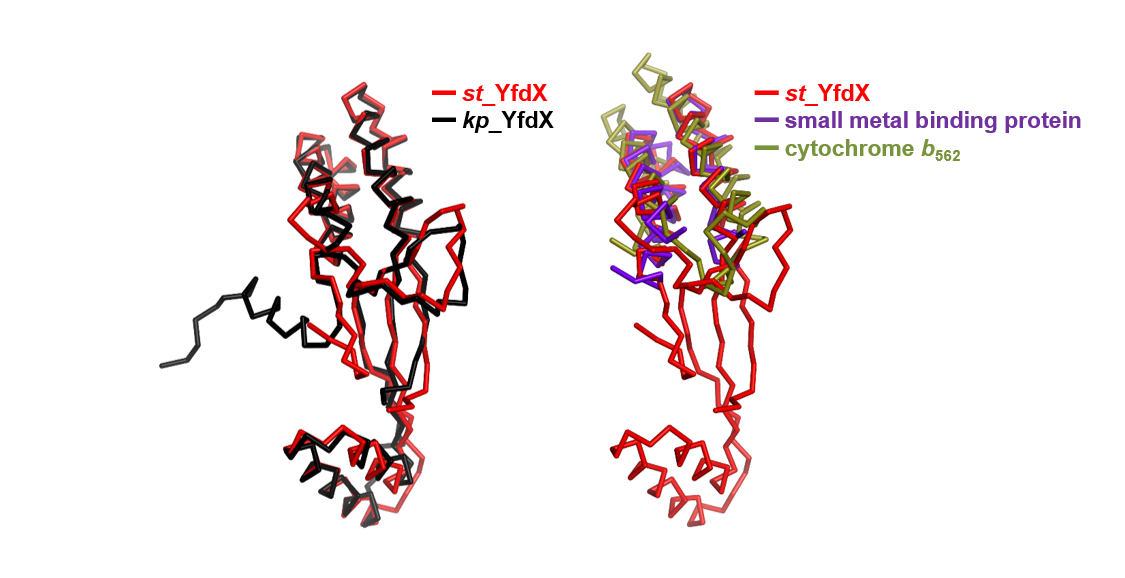


**Supplementary Figure S6. Structural superimposition of *st_*YfdX onto homologous structures**

A monomer of *st_*YfdX is aligned with that of *kp_*YfdX (*left*) and those of *N. europaea* small metal-binding protein and *E. coli* cytochrome *b*_562_ (*right*), which were identified as homologous proteins during a search on the DALI server.

**
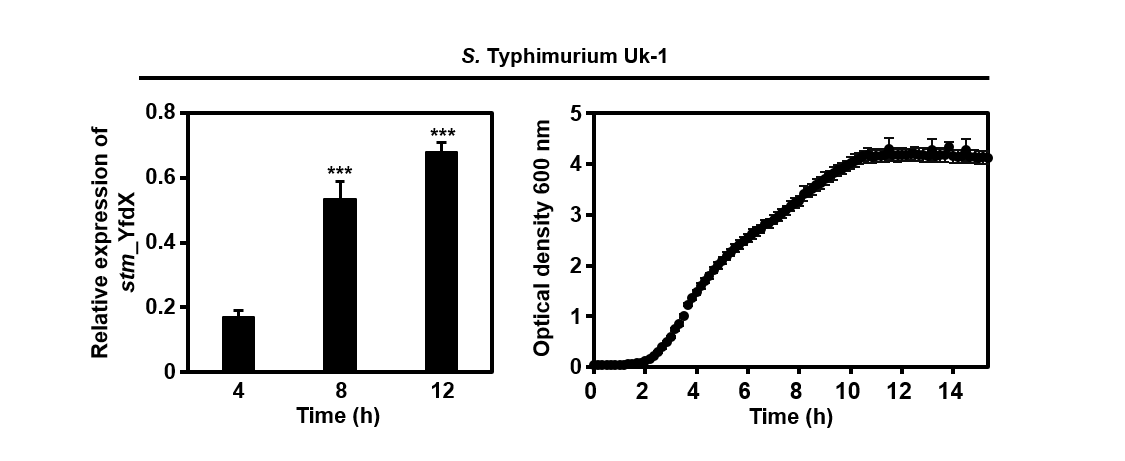
**

**Supplementary Figure S7. Expression levels of *Salmonella* YfdX in live bacteria**

*(Left)* Transcription levels of *stm*_YfdX at the indicated time points were measured by reverse transcription polymerase chain reaction and compared. ns, not significant; ***, P < 0.001 as compared to the transcription level at 4 hours (Student’s *t* test). (*Right*) Growth of *S*. Typhimurium Uk-1 cultured in Luria-Bertani medium. This bacterium entered the exponential phase at ~3 hours and reached the stationary phase at ~11 hours.

**
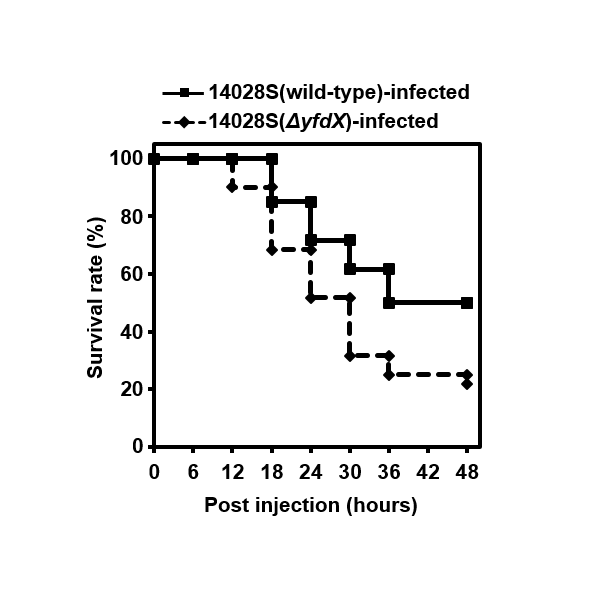
**

**Supplementary Figure S8. YfdX attenuated the virulence of the *S*. Typhimurium 14028S strain**

Survival rates of *G. mellonella* larvae infected by the *S*. Typhimurium 14028S wild-type or Δ*yfdX* strain were measured every 6 hours and compared.
